# Enhancing vaccination uptake through community engagement: evidence from China

**DOI:** 10.1038/s41598-024-61583-5

**Published:** 2024-05-13

**Authors:** Hongyu Guan, Lidong Zhang, Xiangzhe Chen, Yunyun Zhang, Yuxiu Ding, Wenting Liu

**Affiliations:** 1 Center for Experimental Economics in Education, Shaanxi Normal University, Xian, 710119 China; 2 College of Economics, Xi’an University of Finance and Economics, Xi’an, 710100 Shaanxi China

**Keywords:** Health services, Public health

## Abstract

With growing recognition of the importance of community engagement in addressing public health challenges, its role in promoting healthy behaviors and preventing infectious diseases has gained attention. However, vaccination coverage remains a significant concern in many developing countries. While previous studies have linked community engagement to positive health outcomes, there is a gap in understanding its influence on individual vaccination choices, particularly in the context of developing countries. Utilizing data from the 2021 Chinese General Social Survey (CGSS), this study examines the impact of community engagement on COVID-19 and flu vaccination uptake among 7281 individuals. Community engagement, measured by community vaccination notifications, serves as the key independent variable. The study employs Ordinary Least Squares (OLS) regression and Propensity Score Matching (PSM) methods to analyze the relationship between community engagement and vaccination behavior. The analysis reveals a positive association between community engagement and vaccination rates. Specifically, individuals receiving notifications were more likely to get the COVID-19 vaccine compared to non-recipients (vaccination rates: 100% vs. 53.3%), and flu vaccination rates were also significantly higher among those notified (2.7% vs. 1.9%). Mechanism analysis suggests that individuals receiving community notifications are more aware of the benefits of vaccination, leading to higher vaccination rates among this group. This study underscores the effectiveness of community engagement strategies in promoting positive vaccination behavior among individuals in China. By enhancing awareness and trust in immunization, community engagement initiatives play a crucial role in shaping health behaviors and improving vaccination uptake. These findings emphasize the importance of integrating community engagement approaches into public health interventions to address vaccination challenges.

## Introduction

Chronic diseases such as hypertension, obesity, and diabetes, coupled with infectious diseases and child health, pose significant public health challenges, profoundly impacting lives^1–3^. Addressing these multifaceted issues solely through medical interventions is inadequate due to their complexity. Therefore, incorporating social and behavioral approaches is crucial to ensuring better access to healthcare. Community engagement emerges as a critical strategy in this regard, facilitating reciprocal information exchange between community members and the healthcare sector, and gaining widespread recognition from organizations like the World Health Organization^4,5^.

In developing countries, where healthcare disparities are pronounced, community engagement strategies play a crucial role in enhancing healthcare and alleviating stress on citizens' health and survival^6^. China, with its vast population, has notably embraced community engagement strategies, evident in the extensive network of community health service centers and stations established by the end of 2020, providing comprehensive coverage to urban and rural communities^7^.

In terms of implementation outcomes, community engagement strategies have been highly successful in developing countries, notably in managing infectious and chronic diseases. For instance, in combating infectious diseases like COVID-19, community-based precision public health management, including nucleic acid testing and mask promotion, has proven effective^8^. Community engagement has also demonstrated positive impacts on healthcare management across various domains, such as reducing blood pressure and blood sugar^9^, reducing mortality rates among patients with HBV infections^10^, and effective screening for diabetes^11^.

Vaccination management is integral to community health, with its significance magnified in the aftermath of the COVID-19 pandemic^12^. However, developing nations continue to struggle with persistently low vaccination rates. As of the end of 2022, Sudan's COVID-19 vaccination rate stands at a mere 13%, Syria at 18%, and Chad significantly lags behind with only a 2% vaccination rate^13^. While China, as the world's most populous developing country, has achieved commendable COVID-19 vaccination rates, coverage for many other vaccines remains suboptimal. In particular, influenza vaccination rates in China have remained disconcertingly low, with only 23.2% coverage among various population groups on the mainland in 2018^14^. Moreover, from 2011 to 2021, China's adult hepatitis B virus vaccination rate stood at a mere 26.27%^15^. Addressing the challenge of increasing vaccination coverage in developing countries is a pressing matter requiring urgent attention.

Despite numerous global case studies showcasing the effectiveness of community engagement strategies in vaccination promotion, limited research has explored their impact in developing nations and the underlying mechanisms. This study aims to address this gap by focusing on increasing vaccination rates and providing empirical evidence of community engagement's health benefits. Using data from the Chinese General Social Survey (CGSS2021) and focusing on COVID-19 and flu vaccines, our study aims to achieve three specific objectives. Firstly, we aim to compare vaccination rates between communities with high and low levels of community engagement to understand the correlation with higher vaccination rates. Secondly, we seek to investigate the influence of community engagement on individual vaccination decisions using multiple regression analysis while controlling for other variables. Lastly, our study explores potential mechanisms through which community engagement affects vaccination decisions, aiming to identify strategies through which community engagement can be leveraged to enhance vaccination rates.

## Results

### COVID-19 vaccination, flu vaccination, and background characteristics

Table 1 summarizes the characteristics of the variables across all samples, as well as a breakdown based on whether the community has notified residents of vaccination. Across all samples, approximately 45.8% of the individuals were male, the average age was 51.3 years, 93% identified as Han Chinese, and the majority (75.1%) of respondents were married. Moreover, the survey respondents reported good health, with an average self-reported health score of 3.47. Geographically, the distribution of the overall sample's residential areas indicates that 46.1% lived in the eastern region, 33.3% in the central region, and 20.6% in the western region.

**Table 1:** Summary statistics of background characteristics.

Variable, mean (SD)	Full sample	Non community engagement	Community engagement	T-test
				Difference
	(1)	(2)	(3)	(2)–(3)
Panel A: outcome variables				
1. COVID-19 vaccination (1 = yes, 0 = no)	0.731 (0.443)	0.533 (0.499)	1.000 (0.000)	− 0.467***
2. Flu vaccination (1 = yes, 0 = no)	0.022 (0.148)	0.019 (0.136)	0.027 (0.163)	− 0.008**
Panel B: personal characters				
3. Gender (1 = male, 0 = female)	0.458 (0.498)	0.470 (0.499)	0.442 (0.497)	0.029**
4. Age (years)	51.302 (17.221)	50.750 (18.702)	52.053 (14.949)	− 1.303***
5. Ethic (1 = Han, 0 = other)	0.929 (0.258)	0.937 (0.243)	0.917 (0.276)	0.020***
6. Education				
Primary school and below	0.330 (0.471)	0.294 (0.455)	0.382 (0.486)	− 0.088***
Middle school	0.287 (0.452)	0.256 (0.437)	0.328 (0.470)	− 0.072***
High school and above	0.382 (0.486)	0.450 (0.498)	0290 (0.454)	0.160***
7. Household wealth				
Poor	0.331 (0.471)	0.312 (0.463)	0.357 (0.479)	− 0.046***
Average	0.317 (0.465)	0.287 (0.453)	0.3578 (0.479)	− 0.071***
Rich	0.352 (0.478)	0.401 (0.490)	0.285 (0.452)	0.116***
8. Marriage (1 = yes, 0 = no)	0.751 (0.433)	0.707 (0.455)	0.810 (0.392)	− 0.103***
9. Health (score 1–5)	3.470 (1.085)	3.457 (1.098)	3.487 (1.068)	− 0.030
10. Living area				
Eastern China	0.461 (0.498)	0.501 (0.500)	0.406 (0.491)	0.095***
Central China	0.333 (0.471)	0.324 (0.468)	0.345 (0.475)	− 0.021*
Western China	0.206 (0.405)	0.175 (0.380)	0.249 (0.432)	− 0.074***
N	7281	4192	3089	

Means and t-test differences for each variable between the two sets of samples.

***p < 0.01, **p < 0.05, *p < 0.1.

The overall COVID-19 vaccination rate across all samples was 73.1%. Notably, individuals receiving community notification exhibited a significantly higher vaccination rate (100%) than those not receiving community notification (53.3%). This difference was statistically significant (p < 0.01). Meanwhile, the flu vaccination rates were notably low, with a mere 2.2% across all samples. In this context, individuals receiving community notification displayed a higher flu vaccination rate (2.7%) than individuals not receiving community notification (1.9%), with 5% significance (p = 0.017).

The impact of community engagement on individual vaccination rates is analyzed in Table 2. In Column (1), it is evident that community engagement significantly enhances COVID-19 vaccination rates (p < 0.01). Notably, individuals who received community notification exhibited a substantially higher COVID-19 vaccination rate, with an increase of 47 percentage points compared to those who did not receive such notification (Table 2, row 1). Moreover, various demographic and socio-economic factors were found to influence COVID-19 vaccination rates significantly. Specifically, females, younger individuals, those with higher education levels, greater household wealth (moderate or rich), married individuals, those in better health conditions, and residents in central and western China showed elevated rates of COVID-19 vaccination (All p-values < 0.01). Additionally, ethnicity also played a significant role, with members of ethnic minorities displaying higher vaccination rates (p = 0.022).

**Table 2:** Effect of community engagement on COVID-19 vaccination and flu vaccination (OLS).

Variable	(1) COVID-19 vaccination	(2) Flu vaccination
1. community engagement	0.470*** (0.008)	0.009** (0.004)
2. Gender	− 0.024*** (0.008)	− 0.009** (0.004)
3. Age	− 0.006*** (0.000)	0.000** (0.000)
4. Ethic	− 0.036** (0.016)	− 0.007 (0.007)
5. Education		
Middleschool	0.032*** (0.011)	0.007 (0.005)
Highschool	0.087*** (0.012)	0.010* (0.005)
6. Household wealth		
Average	0.062*** (0.010)	0.006 (0.004)
Rich	0.039*** (0.011)	0.010** (0.005)
7. Marriage	0.037*** (0.010)	− 0.001 (0.004)
8. Health	0.038*** (0.004)	0.003 (0.002)
9. Livingarea		
Middle China	0.059*** (0.009)	0.005 (0.004)
West China	0.134*** (0.011)	0.007 (0.005)
10. Constant	0.616*** (0.030)	− 0.006 (0.013)
11. Observations	7281	7281
12. R-squared	0.400	0.003
13. Mean without community engagement population	0.533	0.019

Coefficients were estimated by OLS regression. Standard errors in parentheses; ***p < 0.01, **p < 0.05.

In Column (2), similar positive effects of community engagement are observed on flu vaccination rates (p = 0.014). Individuals who received community notification showed a modest but significant increase of 0.9 percentage points in flu vaccination rates compared to those who did not receive such notification (Table 2, row 1). Furthermore, demographic and socio-economic factors were identified as influencers of flu vaccination rates. Specifically, females, older individuals, and those with higher household wealth (rich) exhibited elevated flu vaccination rates (The p-values were 0.014, 0.039 and 0.035, respectively). Additionally, educational attainment was associated with flu vaccination rates, with individuals having a high school education showing higher vaccination rates (p = 0.061).

### PSM result

The study thus far has revealed a correlation between community engagement and both COVID-19 vaccination and flu vaccination. To ensure the robustness of our findings, we rigorously validate our results using the propensity score matching (PSM) method. Given that community engagement is not a random event and can be influenced by various factors such as age, household wealth, and regions, there is a potential for bias in the estimates^16–18^. If left unaddressed, this bias could significantly skew the distribution of relevant eigenvalues. Hence, employing the propensity score matching method becomes crucial for accurately estimating the impact of community engagement. We conduct one-to-one matching, caliper matching, and kernel matching to enhance the reliability of our analyses. Balance tests for the matched samples are shown in Supplementary Table A1.

Table 3 presents the PSM results. Column (1) depicts the effects of community engagement on COVID-19 vaccination. The Average Treatment Effect on the Treated (ATT) value obtained through one-to-one matching was 0.466 (p < 0.01). This indicates that individuals receiving community notification exhibited a 46.6% higher rate of COVID-19 vaccination compared to those not receiving such notification. The ATT values obtained through caliper matching and kernel matching were 0.464 (p < 0.01) and 0.464 (p < 0.01) respectively, aligning with the results of one-to-one matching.

**Table 3:** Effect of community engagement on COVID-19 vaccination and flu vaccination (PSM).

Variable	(1) COVID-19 vaccination	(2) flu vaccination
1. One to one matching	0.466*** (0.016)	0.007** (0.005)
2. Caliper matching	0.464*** (0.004)	0.010** (0.005)
3. Kernel matching	0.464*** (0.009)	0.010* (0.005)

The average treatment effect on the treated (ATT) estimated by one-to-one matching, caliper matching, and kernel matching corresponded to the first, second, and third rows respectively. Standard errors in parentheses; ***p < 0.01, **p < 0.05, *p < 0.1.

Column (2) illustrates the effects of community engagement on flu vaccination. The ATT value derived through one-to-one matching was 0.007 (p = 0.011). Similarly, the ATT values obtained through caliper matching and kernel matching were 0.01 (p = 0.016) and 0.01 (p = 0.078) respectively. These findings collectively indicate that individuals receiving community notification have higher rates of flu vaccination compared to those not receiving such notification.

### Mechanisms analysis

In this subsection, we delve into potential mechanisms to gain a deeper understanding of how increasing awareness about vaccination through community notification influences individuals' vaccination behaviors. Drawing insights from existing literature, the recognition of vaccination benefits emerges as a pivotal driver of vaccine uptake^19^. We embarked on an exploration of the link between community engagement and individuals' perceptions regarding the merits of vaccination, encompassing notions such as the supremacy of vaccination benefits over drawbacks and the establishment of immunity through vaccination. We used the following two dummy variables: “Believed that the benefits of vaccination outweigh the disadvantages” and “Believed that vaccination gives immunity more than disease”. The above two variables were constructed from the answers to the following two questions in the CGSS2021 questionnaire, which were “Whether you agree that the benefits of vaccination outweigh the disadvantages” and “Do you agree that immunity through vaccination is better than disease”. Table 4 presents two possible patterns for increasing awareness about vaccination through community notification.

**Table 4:** Mechanism analysis.

Variable	Believe that the benefits of vaccination outweigh the disadvantages	Believed that vaccination gives immunity more than disease
1. Community engagement	0.018* (0.011)	0.002 (0.011)
2. Control variable	YES	YES
3. Mean in without community engagement population	0.266	0.286
N	7281	7281

Coefficients were estimated by OLS regression. Standard errors in parentheses; *p < 0.1.

As outlined in Table 4, Column 1, our findings reveal that community engagement is particularly beneficial for individuals in recognizing the superior advantages of vaccination (p = 0.089). By fostering an accurate understanding of this health concept, communities informing residents about vaccination can effectively promote vaccine adoption. However, we did not find a significant correlation between community engagement and the perception that belief in vaccination improves immunity (Table 4, Column 2).

### Heterogeneous analysis

We extend our analysis to encompass the heterogeneous effects of community engagement by incorporating interaction terms. As evidenced in Table 5, our investigation reveals clear indications of diverse effects across various demographic and household attributes within our surveyed cohort. These attributes include gender, age, education level, household wealth, health status, and geographic location.

**Table 5:** Heterogeneous effect.

Variable	(1) COVID-19 vaccination	(2) flu vaccination
1. Community engagement × gender	0.029* (0.016)	− 0.014** (0.007)
2. Community engagement × age	0.012*** (0.000)	0.001** (0.000)
3. Community engagement × ethic	0.023 (0.031)	0.006 (0.014)
4. Education		
Community engagement × middleschool	− 0.174*** (0.020)	− 0.003 (0.009)
Community engagement × highschool	− 0.294*** (0.020)	− 0.017** (0.009)
5. Household wealth		
Community engagement × average	− 0.141*** (0.020)	0.007 (0.009)
Community engagement × rich	− 0.199*** (0.020)	0.003 (0.009)
6. Community engagement × marriage	− 0.022 (0.020)	− 0.001 (0.008)
7. Community engagement × health	− 0.112*** (0.007)	− 0.001 (0.003)
8. Livingarea		
Community engagement × Middle China	− 0.074*** (0.019)	− 0.006 (0.008)
Community engagement × West China	− 0.161*** (0.022)	0.007 (0.009)

Coefficients were estimated by OLS regression. The results reported only the coefficients of the interaction terms between community engagement and each of the characteristic variables. Standard errors in parentheses; ***p < 0.01, **p < 0.05, *p < 0.1.

In Column (1), we examine the effect of community engagement on COVID-19 vaccination among individuals with different characteristics. Our findings suggest that under community engagement for vaccination, COVID-19 vaccination rates among the elderly are 1.2 percentage points higher than among the youth (p < 0.01). Moreover, individuals with lower levels of education exhibit vaccination rates that are 17 to 29 percentage points higher compared to those with higher education levels (p < 0.01). Additionally, vaccination rates for individuals with relatively poor health are 11 percentage points higher than those with relatively good health (p < 0.01). Regarding household wealth, vaccination rates among the economically disadvantaged are 14 to 20 percentage points higher than among the affluent (p < 0.01). Geographically, vaccination rates among individuals living in eastern China are 7 to 16 percentage points higher than those in central and western China (p < 0.01). Overall, community engagement appears to increase the likelihood of COVID-19 vaccination, particularly among older individuals, those with lower education levels, poorer household wealth status, less healthy individuals, and residents of eastern China.

In Column (2), we investigate the effect of community engagement on flu vaccination among individuals with different characteristics. Our results indicate that under community engagement for vaccination, flu vaccination rates among females are 1.4 percentage points higher than among males (p = 0.05). Additionally, vaccination rates among the elderly are 0.1 percentage points higher than among the youth (p = 0.022). Similarly to COVID-19 vaccination, individuals with lower levels of education show flu vaccination rates that are 1.7 percentage points higher compared to those with higher education levels (p = 0.043). Overall, community engagement appears to increase the likelihood of flu vaccination, particularly among females, older individuals, and those with lower education levels.

## Discussion

In the context of China, the quantification of the impact of community engagement strategies on individual vaccinations remains an understudied area. Our empirical study, utilizing a nationally representative dataset from China, not only provides evidence but also enriches our understanding of how community engagement influences individual vaccination behaviors in developing countries. By examining COVID-19 and influenza vaccinations as indicators, our research unveils a positive correlation between community notification efforts and both types of individual immunizations. This finding aligns with prior research on the influence of community engagement strategies on personal health outcomes^20^.

We extensively explored the potential mechanisms underlying the influence of community engagement on individuals' vaccination behaviors in our study. Our findings suggest that individuals who received community notifications demonstrated higher vaccination rates, likely attributed to these notifications enhancing their perception of vaccination's value and persuading them of its benefits. This aligns with the conclusions of several studies indicating that community interventions can alter public perceptions of public health services, thereby fostering better acceptance of appropriate measures by residents^21–23^. According to the authority orientation theory, individuals are more inclined to adhere to rules when they perceive authority as reliable and trustworthy^24^. The significance of community services, particularly during the COVID-19 pandemic, has been greatly emphasized. To a considerable extent, the information disseminated by communities and the measures they implement can be perceived by residents as authoritative. Thus, community engagement effectively enhances people's understanding of healthy habits and cultivates trust in immunization, thereby increasing their willingness to comply with vaccination recommendations.

The heterogeneity analysis reveals that community engagement has a more pronounced impact on vaccination behavior among the elderly and those with lower education levels. Elderly individuals, who rely more on community notifications due to lower usage of electronic devices for health information^25^, and individuals with lower education levels, who are more reliant on community promotions^26^, exhibit heightened responsiveness to such efforts. Specifically, regarding COVID-19 vaccination, community engagement appears particularly effective for groups with poorer health, lower household wealth, and residing in eastern China. Individuals in poor health are more receptive to community efforts, while those with lower household wealth may face greater challenges in managing disease-related implications, making them particularly sensitive to community notification behaviors^27^. The robust implementation of community services in eastern China fosters high resident satisfaction and increased responsiveness to community notices^28^. This underscores the importance of tailoring community engagement strategies to demographic and socioeconomic characteristics to effectively promote vaccination uptake, especially in vulnerable populations.

This study presents notable strengths and valuable contributions. It tries to offer causal insights into the impact of community engagement on vaccination, thereby enriching the existing literature. Furthermore, it holds practical implications for immunization policies in developing countries, facilitating efforts to bolster vaccination rates by integrating healthcare with community service provision. Lastly, it sheds light on how community engagement strategies, such as notification, influence individual vaccination behavior, potentially enhancing public awareness of community service management in the future.

However, this study has certain limitations that warrant acknowledgment. Firstly, due to data constraints, we were unable to explore the nuanced effects of the frequency and intensity of community notification implementation on vaccination rates. Future studies should delve deeper into this aspect to gain a comprehensive understanding of how variations in notification strategies may impact vaccination behaviors. Secondly, it's important to recognize the potential influence of self-report bias on our findings, as vaccination status relies on self-reported information, which may be subject to recall bias or social desirability bias. While efforts were made to mitigate bias through rigorous data collection and analysis procedures, the inherent limitations of self-reporting must be acknowledged. However, considering the severity of the COVID-19 pandemic in 2021, along with the Chinese government's stringent epidemic prevention and control policies, and the high level of community emphasis on epidemic management, coupled with the 82.5% nationwide full vaccination coverage rate announced by the National Health Commission in December 2021^29^, which exceeds our sample group's vaccination rate of 73%, so we believe that our data is relatively close to the actual situation. Therefore, Future studies could benefit from incorporating objective measures of vaccination status, such as medical records or immunization registries, to validate self-reported data and enhance the robustness of study findings. Lastly, our study's exclusive focus on China may restrict the applicability of our findings to other contexts due to cultural, social, and economic variations. Caution is advised when extrapolating our results, and future research should aim to replicate our findings across diverse populations to ensure the broader effectiveness of community engagement strategies in improving vaccination uptake.

To translate these findings into actionable policies and practices, policymakers should consider tailored community engagement campaigns leveraging various communication channels to raise awareness about the benefits of COVID-19 and flu vaccinations. Strengthening the capacity of community health workers and fostering collaborative partnerships between government health agencies and local organizations are essential steps in enhancing vaccination uptake. Additionally, conducting comprehensive vaccine education and promotional campaigns, leveraging community platforms, and organizing interactive events will effectively raise awareness among residents and foster greater trust and acceptance towards vaccines. Personalized vaccination programs and services should be tailored to suit the unique characteristics of different communities and the specific needs of their residents. Special support and incentives should be provided for vulnerable groups, such as the elderly, individuals with limited education, and low-income families.

In addition to the current study's findings, future research should explore the differential impacts of various community engagement strategies, such as social media campaigns or targeted outreach programs, on vaccination behavior to identify the most effective approaches. Longitudinal studies investigating the sustained effects of community engagement initiatives on vaccination uptake and public health outcomes would further enhance our understanding of their effectiveness over time. Furthermore, examining the role of digital health technologies, such as mobile applications or online platforms, in facilitating community engagement and addressing vaccine hesitancy among underserved populations warrants further investigation.

Future research endeavors should expand upon the findings of this study by investigating the varying impacts of diverse community engagement strategies, such as social media campaigns or targeted outreach programs, on vaccination behavior. Longitudinal studies exploring the sustained effects of community engagement initiatives on vaccination uptake and public health outcomes would provide invaluable insights into their effectiveness over time. Moreover, the COVID-19 pandemic has spotlighted the significance of digital health technologies, notably Digital Contact Tracing Applications (DCTAs), which have garnered substantial attention and usage^30^. Examining the role of digital health technologies, including DCTAs, mobile applications, or online platforms, in tandem with community engagement could significantly enhance public health interventions and address vaccination challenges, particularly among underserved populations. Furthermore, in developing countries where access to vaccines may be limited, community engagement may exert influence on a broader spectrum of health behaviors, such as handwashing, which are pivotal in combating infectious diseases. Therefore, future research should delve deeper into the impact of community engagement on such behaviors and explore diverse interventions to promote public health practices.

## Conclusion

In conclusion, this study underscores the importance of community engagement in promoting individual vaccination practices and highlights the potential for tailored campaigns and strengthened partnerships to improve vaccination uptake. Moving forward, expanding research efforts beyond China to encompass other developing countries will be crucial for advancing our understanding of community engagement strategies' broader applicability in promoting public health initiatives worldwide.

## Method

### Data

This research utilizes data from the 2021 Chinese General Social Survey (CGSS), conducted by the China Survey and Data Center of Renmin University of China. CGSS, initiated in 2003, is a comprehensive and continuous academic survey project, widely recognized for its authoritative database with extensive samples, broad coverage, and diverse content. The survey process is meticulously executed by the CGSS Project Team, collaborating with academic institutions across the country. During the sampling process, the team employs a multi-stage stratified PPS sampling method, ensuring a representative selection of respondents. The surveyors, equipped with professional expertise in statistical surveys, guide the respondents through the questionnaire, collecting data that are both scientific and representative. The 2021 dataset, comprising 8,148 valid samples, provides multi-level data on social, family, and individual aspects, adequately catering to the research requirements regarding community engagement for vaccination. Consequently, 7,281 samples were retained for this study.

In this paper, CGSS2021 was chosen for several reasons. Firstly, the survey boasts a comprehensive sample covering 19 provinces, autonomous regions, and municipalities directly under the central government. This extensive coverage ensures a substantial amount of valid data, making it an ideal choice for our research. Secondly, CGSS2021's questionnaire includes a new section on COVID-19 vaccination status, which greatly enhances the relevance and value of our study, particularly in exploring community engagement for vaccination. Finally, the survey offers a rich array of variables, including community engagement, vaccination status, and individual perceptions of vaccination, among numerous other factors central to our investigation.

### Ethics approval and consent to participate

Research ethics approval for data collection in the Chinese General Social Survey (CGSS) dataset was granted by the Institutional Review Board of Renmin University, PR China. The survey was conducted in accordance with the ethical guidelines laid down in the Declaration of Helsinki. All participants provided written informed consent before taking part in the survey.

### Variables

Our analysis focuses on two dependent variables: COVID-19 vaccination and flu vaccination. For COVID-19 vaccination, respondents were asked, "Currently, whether you are vaccinated against COVID-19 or not?" We define an individual as vaccinated if they answered "YES," constructing a dummy variable (YES = 1). Similarly, for flu vaccination, respondents were asked, "Did you get the flu vaccine last year?" Those answering "YES" were considered vaccinated, with another dummy variable constructed accordingly (YES = 1).

The primary independent variable is community engagement, operationalized based on the presence of vaccine notification activities. Respondents were asked, "Were you informed about vaccinations by your community?" A positive response indicated active mobilization by the community for vaccination, resulting in a dummy variable (YES = 1).

It needs to be clarified that the term "community," as discussed in our paper, broadly refers to a geographic area where residents share social connections. These areas are often managed by local government authorities or community residents' committees worldwide, with varying degrees of political involvement. Particularly in China, communities are pivotal, especially amidst the COVID-19 pandemic, serving as geographic areas with a quasi-administrative role, where residents coexist and engage in various activities. They are often overseen by local government authorities or community residents' committees, with varying levels of political involvement.

In addition to community engagement, demographic and family characteristics are controlled to avoid bias from omitted variables. These include gender (male = 1), age (years), ethic (han = 1), educational attainment, family economic condition, marriage (married = 1), health status (higher scores indicate better health status: very unhealthy = 1, relatively unhealthy = 2, healthy = 3, relatively healthy = 4, very healthy = 5), and living area (taking "East China" as a reference, two dummy variables were constructed: "Central China" and "West China"). Descriptive statistics for these variables are presented in Table 1.

### Statistics analysis

This study initially provides descriptive statistics of individual and family background characteristics. Furthermore, we conduct group comparisons of these characteristics between community engagement and non-community engagement using sample t-tests to discern any mean differences between the two groups.

To address the primary focus of this study, we employ ordinary least squares (OLS) regression models to estimate the impact of community engagement on COVID-19 vaccination and flu vaccination behavior:

1 $${{\text{Y}}}_{{\text{i}}}=\mathrm{\alpha }+{\upbeta }_{1}{\text{Community engagement}}\,_{{\text{i}}}+{\upbeta }_{2}{{\text{X}}}_{{\text{i}}}+{\upvarepsilon }\,_{{\text{i}}}$$

Here, ${{\text{Y}}}_{{\text{i}}}$ represents a binary indicator of individual *i*'s COVID-19 vaccination or flu vaccination behavior, while ${\text{Community engagement}}\,_{{\text{i}}}$ is a dummy variable indicating whether the community has notified residents of vaccination. ${{\text{X}}}_{{\text{i}}}$ denotes a vector of baseline variables correlated with an individual’s vaccination behavior, encompassing demographic factors (gender, age, ethnicity, educational attainment), family factors (family economic condition, marriage, health status, and living area), and vaccination behavior-related factors (health status). ${\upvarepsilon }_{{\text{i}}}$ represents a random error term. In the regression models, we adjust standard errors for clustering at the individual level using the cluster-corrected Huber-White estimator.

Our analysis employed propensity score matching (PSM)^31,32^, a method designed to mitigate selection bias and provide a robust estimation of treatment effects in situations where systematic differences between groups are non-random^33^. The propensity score, derived from individual covariates, quantifies the likelihood of an individual receiving notifications. Matching techniques then pair individuals who did not receive notifications with similar counterparts who did, based on their baseline characteristics. Covariates utilized in creating propensity scores encompassed gender, age, ethnicity, educational attainment, family socioeconomic status, marital status, health status, and residential area.

All analyses are conducted using Stata 15.0 (Stata Corp., Texas, USA). Two-sided tests are employed, and significance is determined at *p* < 0.1.

## Supplementary Information

Supplementary Table 1.

## Data Availability

The datasets used and/or analyzed during the current study are available from the corresponding author on reasonable request.
