# Supplementary material for: Enhancing vaccination uptake through community engagement: evidence from China
Source: Sci Rep. 2024 May 13;14:10845. doi: 10.1038/s41598-024-61583-5 (PMC11636859; doi:10.1038/s41598-024-61583-5)
Supplement: Supplementary file 1 — Supplementary Table 1. [file 41598_2024_61583_MOESM1_ESM.pdf]

Title

Enhancing Vaccination Uptake Through Community Engagement: Evidence from China

Authors

Hongyu Guan[1], Lidong Zhang[1], Xiangzhe Chen[1],Yunyun Zhang[2*], Yuxiu Ding[1], Wenting Liu[1]

[1]Center for Experimental Economics in Education, Shaanxi Normal University, Xian, 710119, China

[2]College of Economics, Xi'an University of Finance and Economics, Xi'an, 710100, China

Supplementary Table A1. Balance test of the matched sample

VARIABLES	(1)Non community engagement (mean)	(2)Community engagement (mean)	(3)T-test Difference
**Panel A: one to one matching**			
gender	0.442	0.426	1.21
age	52.067	52.6	-1.33
ethic	0.918	0.929	-1.72*
middleschool	0.328	0.328	0.03
highschool	0.290	0.290	0.03
average	0.358	0.346	0.99
rich	0.285	0.289	-0.31
marriage	0.810	0.831	-2.16**
health	3.486	3.441	1.69
middlechina	0.345	0.356	-0.91
westchina	0.248	0.246	0.24
**Panel B: caliper matching**			
gender	0.442	0.439	0.13
age	52.067	52.227	-0.39
ethic	0.917	0.921	-0.51
middleschool	0.328	0.334	-0.47
highschool	0.290	0.290	-0.03
average	0.357	0.362	-0.40
rich	0.285	0.289	-0.33
marriage	0.809	0.810	-0.10
health	3.485	3.494	-0.32
middlechina	0.345	0.342	0.23
westchina	0.248	0.250	-0.19

**Panel C: kernel matching**			
gender	0.441	0.441	0.04
age	52.067	52.261	-0.47
ethic	0.917	0.924	-0.93
middleschool	0.328	0.331	-0.26
highschool	0.290	0.294	-0.37
average	0.357	0.358	-0.10
rich	0.285	0.289	-0.32
marriage	0.809	0.808	0.10
health	3.485	3.485	0.01
middlechina	0.345	0.346	-0.12
westchina	0.248	0.238	0.89

Note: Means and t-test differences for each variable between the two sets of matched samples. *** p<0.01, ** p<0.05, * p<0.1
